# Trace and Essential Elements Analysis in *Cymbopogon citratus* (DC.) Stapf Samples by Graphite Furnace-Atomic Absorption Spectroscopy and Its Health Concern

**DOI:** 10.1155/2014/690758

**Published:** 2014-12-01

**Authors:** Jasha Momo H. Anal

**Affiliations:** ^1^Elemental Analysis Section, Sophisticated Analytical Instrument Facility (SAIF), North Eastern Hill University, Shillong, Meghalaya 793022, India; ^2^Center for Advanced Studies in Chemistry, North Eastern Hill University, Shillong, Meghalaya 793022, India

## Abstract

*Cymbopogon citratus* (DC.) Stapf commonly known as lemon grass is used extensively as green tea and even as herbal tea ingredient across the world. Plants have the ability to uptake metals as nutrient from the soil and its environment which are so essential for their physiological and biochemical growth. Concentrations of these twelve trace elements, namely, Mg, Ca, Cr, Mn, Fe, Ni, Cu, Zn, Mo, As, Cd, and Pb, are analysed by graphite furnace-atomic absorption spectroscopy (GF-AAS) and are compared with the permissible limits of FAO/WHO, ICMR, and NIH, USA, which are found to be within permissible limits. Toxic metals like As, Cd, and Pb, analysed are within the tolerable daily diet limit and at low concentration.

## 1. Introduction

The genus* Cymbopogon* belongs to one of the most important essential oil yielding families of the Poaceae and comprises nearly 140 species that are widely distributed in semitemperate to tropical regions of Asia, Africa, and America. Approximately 45 species have been reported to occur in India. The* Cymbopogon* species that produce volatile oils are called aromatic grasses [1].* Cymbopogon citratus* (DC.) Stapf is native from the southwest Asia and now it grows spontaneously around the world, mainly in the tropical and savannah regions [2]. Commonly, it is known as lemongrass, which is a perennial herb, commercially cultivated in Guatemala, India, China, Paraguay, Sri Lanka, and also some areas of Pakistan [3].

Green tea is a rich source of polyphenols, which are antioxidants in nature. Among the various types of tea, green tea contains a relatively high level of polyphenols, which consist of avanol monomers (avan-3-ols), also referred to as catechins [4]. Natural antioxidants, such as polyphenols from green tea extracts, have recently attracted considerable attention for preventing oxidative stress-related diseases including cancers, cardiovascular diseases, and degenerative diseases [5]. Tea plays a major role in terms of the intake of a number of nutritional trace elements in humans. Besides many essential elements required for human health, some toxic elements may also be present in the parts used for the tea. This could be due to contaminated soil, pesticides applications, fertilizers, or industrial activities. Due to the significant amount of tea consumed, it is important to know the toxic metal contents [6].

## 2. Materials and Methods 

### 2.1. Reagents and Chemicals

All chemicals used were of analytical grade purchased from S.D. Fine-Chem. Ltd. and Qualigens, Fischer Scientific, India. The ultrapure deionized water (Millipore S.A., France) was used for the preparation of standards and modifier solutions. The stock standard solutions were purchased from Sigma Aldrich chemical company for calibration by preparing standard solutions.

### 2.2. Plant Sample Collection

The samples of* Cymbopogon citratus *were collected from the market of Imphal, Manipur, where the plant has been cultivated for commercial purpose and even domestically for various medicinal properties. The species is widely used in making green tea in the region.

### 2.3. Plant Sample Digestion

For the digestion of plant sample, 0.5 g sample(s) was weighed and was digested for 3 h at 85°C with concentrated HNO_3_ : HCl (3 : 1) mixture. Then concentrated HClO_4_ (1 mL) was added to enhance the oxidation properties in the digestion. The solutions were filtered and diluted to 50 mL with distilled water. The blank solution was taken as the same procedure without addition of the sample [7].

### 2.4. Instrumentation

An Analytik Jena AAS Vario-6 Graphite furnace spectrometer furnished with PC-controlled 6-piece lamp turret, where hallow cathode lamps are mounted as line radiator along with a deuterium hallow cathode lamp for compensation of the background absorption and argon gas supply, was used for all of the absorption measurements. The hollow cathode lamps fitted for specific element that has to be analyzed with their respective wavelength and the slit width adjusted accordingly. Signal measurement was done in peak area/peak height and calibration was in linear mode. The sample injection volume is 20 *μ*L. The typical heating program of GF-AAS is drying (injection of the sample into the filter furnace), pyrolysis, atomization, and cleansing. The elements instrumental conditions are given in Table 1.

### 2.5. Data Analysis

Calculation of each heavy metal depends on the laboratory procedure and WinAAS Version 3.10 software (Analytik Jena, Germany). Calibration curves were prepared using a linear curve. Data analysis was performed using SPSS data editor 16.0 and Microsoft Office Excel 2007.

## 3. Results and Discussion

Analysis of the twelve trace elements, namely, Mg, Ca, Cr, Mn, Fe, Ni, Cu, Zn, Mo, As, Cd, and Pb, was performed in triplicate samples of* Cymbopogon citratus* collected from markets of Imphal, Manipur. The elemental level analysed in samples of* Cymbopogon citratus* plant is given in Table 2. Trace and essential elements dietary allowance/intake in human adults was compared with nutrient requirements and recommended dietary allowances for Indians (Indian Council of Medical research (ICMR), 2009), Food and Agricultural Organisation (FAO/WHO) (2004), and Food and Nutrition Board, Institute of Medicine, National Academies, USA, as given in Table 3. Trace elements levels in fresh tea leaves and made tea from different countries had been studied and indicated that among them aluminium (Al), arsenic (As), cadmium (Cd), chromium (Cr), copper (Cu), fluoride (F), manganese (Mn), and nickel (Ni) in different tea infusions ranges were 0.06–16.82 mg L^−1^; trace, 1.53 *μ*g L^−1^; trace, 0.79 *μ*g L^−1^; below detectable limit, 43.2 *μ*g L^−1^, 0.02–40.0 mg L^−1^, 0.2–4.54 mg L^−1^, 0.1–250 mg L^−1^, and BDL—0.16 mg L^−1^, respectively. Besides essential macro- and microelements, experimental studies have demonstrated that the accumulation of significant amount of excess nonessential trace elements in tea leaves may eventually increase the metal body burden in humans [6].

The trace toxic elements have caused main human health problems in several parts of the world and contrasting metabolisms cause relative scale of such incidents. Tolerable daily intake of heavy metals like As, Cd, and Pb in ingested products has been set by FAO/WHO, FDA/USA, and California Standards (CS) as given in Table 4.

### 3.1. Magnesium

The level of this element in this plant samples ranges from 0.764 to 0.0.790 mg/kg. Magnesium functions as a cofactor of many enzymes involved in energy metabolism, protein synthesis, RNA and DNA synthesis, and maintenance of the electrical potential of nervous tissues and cell membranes [8, 9]. In addition, low serum and dietary Mg may be related to the etiologies of cardiovascular disease, hypertension, diabetes, and atherosclerosis in humans [10].

### 3.2. Calcium

Calcium content in the plant samples ranges from 5.157 to 6.012 mg/kg. The element is an essential nutrient that plays a vital role in neuromuscular function, many enzyme-mediated processes, blood clotting if not most, and metabolic processes as well as providing rigidity to the skeleton by virtue of its phosphate salts. Its nonstructural roles require the strict maintenance of ionized calcium concentration in tissue fluids at the expense of the skeleton if necessary and it is therefore the skeleton which is at risk if the supply of calcium falls short of the requirement. Calcium fluxes are also important mediators of hormonal effects on target organs through several intracellular signalling pathways [11].

### 3.3. Chromium

The level of this element in this plant samples ranges from 0.601 to 0.873 mg/kg. Chromium occurs naturally in its combined state and usually not in its free state. The most stable valance states of Cr are Cr(+3) and Cr(+6) found in foods supply, air, water, plants soil, animals, and environments. Several million industrial workers worldwide are potentially exposed to Cr and Cr-containing compounds [12], although native chromium was reported to be found in its trace metallic form (Cr^0^) [13].

Chromium is an essential nutrient that potentiates insulin action and thus influences carbohydrate, lipid, and protein metabolism [14]. However, the role of chromium as cofactor for insulin action is not fully understood as studies from several* in vivo* and* in vitro* studies at the molecular level are ongoing [15].

### 3.4. Manganese

Manganese content in the plant sample ranges from 0.193 to 0.271 mg/kg. Mn is an essential ubiquitous trace element required for normal growth, development, and cellular homeostasis [16]. In addition, it is reported to have a role in neurodegenerative diseases [17].

### 3.5. Iron

The level of this element in this plant samples ranges from 1.644 to 1.984 mg/kg. Fe deficiency is probably the most common nutritional deficiency disorder in the world though it performs the most vital functions in the body. An estimate based on WHO criteria indicated that around 600–700 million people worldwide have marked iron deficiency anaemia, and the bulk of these people live in developing countries. In developed countries, the prevalence of iron deficiency anaemia is much lower and usually varies between 2% and 8%. However, the prevalence of iron deficiency, including both anaemic and nonanaemic subjects, is much higher. In developed countries, for example, an absence of iron stores or subnormal serum ferritin values is found in about 20–30% of women of fertile age. In adolescent girls, the prevalence is even higher [18].

### 3.6. Nickel

Nickel content in the plant sample ranges from 0.046 to 0.105 mg/kg. Ni is essential for the catalytic activity of some plant and bacterial enzymes. It is said to influence iron absorption and metabolism and the hemopoietic process. However, biochemical functions of nickel have not been demonstrated in humans and higher animals. Humans exposed to highly nickel-polluted environments are at higher risk to etiological and pathological effects as it is known to cause lungs and nasal cancers [19].

### 3.7. Copper

Copper content in the plant sample ranges from 0.051 to 0.077 mg/kg. Functional roles for copper is one of the most important in human's life like iron and are found in erythropoietins, myelin formation, modulation of catecholamine metabolism, and antioxidant protection and in the regulation of immune functions and cholesterol and glucose metabolism. Copper is a component of the metalloenzymes that take part in the catecholaminergic pathway-monoamine oxidase, dopamine *β*-hydroxylase, and tyrosine hydroxylase [20].

### 3.8. Zinc

Zinc content in the plant sample ranges from 0.105 to 0.122 mg/kg. Zn is an essential component of a large number of enzymes participating in the synthesis and degradation of carbohydrates, lipids, proteins, and nucleic acids as well as in the metabolism of other micronutrients. Zinc stabilizes the molecular structure of cellular components and membranes and in this way contributes to the maintenance of cell and organ integrity. Furthermore, zinc has an essential role in polynucleotide transcription and thus in the process of genetic expression. Its involvement in such fundamental activities probably accounts for the essentiality of zinc for all life forms [21]. It plays a central role in the immune system, affecting a number of aspects of cellular and humoral immunity [22].

### 3.9. Toxic Heavy Metals and Tolerable Daily Intake

The toxicity and effect of trace heavy metals on human health and the environment have attracted substantial awareness and concern in recent years. Among the heavy metals, lead (Pb), cadmium (Cd), and arsenic (As) are especially toxic and are harmful to humans even at low concentrations. The maximum limit for arsenic was fixed at 3.0 mg/kg, for cadmium was 1.0 mg/kg, and lead was reduced to 0.5 mg/kg. They have an inherent toxicity with a tendency to accumulate in the food chain and a particularly low removal rate through excretion [23]. Acceptable limits for daily consumption of heavy metals in our nutritional intakes should closely be watched and quality assurance standards should be maintained [24]. The accepted standard of heavy metal toxicity for ingested products for various agencies and our experimental results is given in Table 4. Exposure to heavy metals above the permissible level can cause high blood pressure, fatigue, and kidney and neurological disorders. Heavy metals are also known to cause harmful reproductive effects [25].

## 4. Conclusion

Next to water, tea prepared from* Camellia sinensis* is the most popular nonalcoholic beverage consumed by about half earth's human population [26]. Considering the hazard index (HI) of daily tea drinking, it appears that frequently consumed black tea may have adverse effects on the human body because HI values for hazard quotients (As, Cr, Cd, and Pb) are close to 1. HIs established for green teas are about 100 times lower than those for black and oolong teas [27]. In addition, green tea prepared from* Cymbopogon citratus* is fast gaining among the list of nonalcoholic beverage because of its aromatic flavour and its many health beneficial properties. The nutrients intake from various tea beverages is well-known though oral intake of nonessential and toxic elements cannot be ruled out as plants can uptake them from the soil and its environment. From this present study, the trace element concentrations in* Cymbopogon citratus* species found in Manipur popularly used as green tea are within the permissible limits from the samples studies.

## Figures and Tables

**Table 1:** AAS Vario-6 Graphite furnace elements instrumental analytical conditions.

Elements	Wavelength (nm)	Slit width (nm)	Atomization temp. (0°C)	Matrix modifiers	Interference wavelength (nm)	Detection limit (*µ*g L^−1^)
Mg	285.2	0.8	1500–1650	—	—	0.0025
Ca	422.7	1.2	2350–2500	—	—	0.007
Cr	357.9	0.8	2100–2200	NH_4_H_2_PO_4_	Fe 358.1, Nb 358.0	0.1
Mn	279.5	0.2	1600–1650	Mg(NO_3_)_2_ + Pd(NO_3_)_2_	Mg 279.5, Fe 279.5, Pb 280.2	0.014
Fe	248.3	0.2	1850–2050	Mg(NO_3_)_2_	—	0.100
Ni	232.0	0.2	2100–2300	Mg(NO_3_)_2_	—	0.3
Cu	324.8	0.8	1800–1900	—	Ni 324.3, Mn 324.9, Pd 324.3, Ag 324.8, Eu 324.8	0.19
Zn	213.9	0.8	1000–1100	Pd(NO_3_)_2_	Cu 213.9, Te 214.3 As 214.4, Fe 213.6, Fe 213.9	0.002
Mo	313.3	0.8	2500–2600	Mg(NO_3_)_2_	—	0.15
As	193.7	0.0	2050–2250	Pd(NO_3_)_2_	—	0.4
Cd	228.8	0.8	900–1200	NH_4_H_2_PO_4 _+ Mg(NO_3_)_2_	As 228.9 Fe 228.8	0.007
Pb	217.0	0.5	1200–1350	Pd(NO_3_)_2_ + Mg(NO_3_)_2_	Cu 216.5, Fe 216.7, Ni 216.6, Sb 217.6, Pt 216.5	0.08

**Table 2:** Distribution levels (mg/kg ± SD) of elemental contents in the triplicate samples of *Cymbopogon citratus* plant (mean values ± SD).

Element	Sample 1	Sample 2	Sample 3
Mg	0.774 ± 0.001	0.7641 ± 0.002	0.790 ± 0.008
Ca	5.157 ± 0.041	6.012 ± 0.085	5.179 ± 0.081
Cr	0.675 ± 0.141	0.601 ± 0.229	0.873 ± 0.483
Mn	0.271 ± 0.036	0.193 ± 0.010	0.214 ± 0.061
Fe	1.645 ± 0.187	1.644 ± 0.014	1.984 ± 0.069
Ni	0.004 ± 0.006	0.006 ± 0.016	0.010 ± 0.017
Cu	0.064 ± 0.003	0.077 ± 0.003	0.051 ± 0.002
Zn	0.122 ± 0.025	0.108 ± 0.001	0.105 ± 0.002
Mo	0.369 ± 0.021	0.413 ± 0.050	0.403 ± 0.035
As	0.047 ± 0.034	0.087 ± 0.054	0.076 ± 0.051
Cd	0.030 ± 0.001	0.017 ± 0.003	0.025 ± 0.001
Pb	0.014 ± 0.001	0.013 ± 0.002	0.031 ± 0.010

**Table 3:** Trace and essential elements dietary allowance/intake in human adults.

Element	Recommended dietary allowance (RDA), ICMR, 2009	Recommended nutrient intake (RNI), FAO/WHO, 2004	Dietary reference intakes: recommended dietary allowance (RDA), USA
Mg	310 mg/d^*^, 340.0 mg/d^**^	220.0 mg/d	420.0 mg/d^*^, 310.0 mg/d^**^
Ca	600.0 mg/d	750.0–800.0 mg/d	1000.0 mg/d
Cr	33 *μ*g/d	25 *μ*g/d	35.0 *μ*g/d^a^
Mn	5.4–17.0 mg/d	—	2.3 mg/d^a∗∗^ and 1.8 mg^a∗∗^
Fe	17.0 mg/d^*^ and 21.0 mg/d^**^	9.1 mg/d^*^, 26.0 mg/d^**^	8.0 mg/d^*^, 18.0 mg/d^**^
Ni	—	—	—
Cu	1.35 mg/d	—	900.0 *μ*g/d
Zn	10.0–12.0 mg/d	4.2–14.0 mg/d	11.0 mg/d^*^ and 8.0 mg/d^**^
Mo	—	—	45.0 *μ*g/d

^*^For male.  ^**^For female. NE = not established, mg/g = milligram per day, *µ*g/d = microgram per day, and a = adequate intakes (AI).

Nutrient requirements and recommended dietary allowances for Indians. A Report of the expert group of the Indian Council of Medical Research (ICMR), 2009.

Vitamin and mineral requirements in human nutrition: report of a joint FAO/WHO expert consultation,World Health Organization, and Food and Agriculture Organization of the United Nations, 2004.

Dietary reference intakes (DRIs): recommended dietary allowances and adequate intakes, Elements Food and Nutrition Board, Institute of Medicine, National Academies. These reports were accessed via http://www.nap.edu/ on April 1, 2014.

**Table 4:** Tolerable daily intake of heavy metals in ingested products.

Metal	Accepted standards of heavy metal toxicity for ingested products			
	California Standards^#^ (CS)	USP^##^	FDA^###^	Experimental results (mean ± SEM^a^)
Arsenic	10.0 *μ*g	3.0 ppm	—	0.070 ± 0.012
Cadmium	4.1 *μ*g	3.0 ppm	—	0.024 ± 0.004
Lead	0.5 *μ*g	10.0 ppm	75.0 *μ*g	0.019 ± 0.006

^a^SEM = standard error mean; ^#^California Proposition 65 Daily Limits for Heavy Metal Consumption, http://www.oehha.ca.gov/prop65/p65faq.html;^ ##^United States Pharmacopeia (USP) Limit for Nutritional Supplements, USP (http://www.usp.org/); ^###^FDA Tolerable Daily Diet Lead Intake (http://www.fda.gov/).

## References

[1] Rao B. L., Handa S. S., Kaul M. K. (1997). Scope for development of new cultivars of *Cymbopogons* as a source of terpene chemicals. *Supplement to Cultivation and Utilization of Aromatic Plants*.

[2] Shah G., Shri R., Panchal V., Sharma N., Singh B., Mann A. S. (2011). Scientific basis for the therapeutic use of *Cymbopogon citratus*, stapf (Lemon grass). *Journal of Advanced Pharmaceutical Technology and Research*.

[3] Bhan M. K., Pal S., Rao B. L., Dhar A. K., Kang M. S. (2005). GGE biplot analysis of oil yield in lemon grass (*Cymbopogon* spp.). *Journal of New Seeds*.

[4] Chen L., Yang X., Jiao H., Zhao B. (2002). Tea catechins protect against lead-induced cytotoxicity, lipid peroxidation, and membrane fluidity in HepG2 cells. *Toxicological Sciences*.

[5] Ogura R., Ikeda N., Yuki K., Morita O., Saigo K., Blackstock C., Nishiyama N., Kasamatsu T. (2008). Genotoxicity studies on green tea catechin. *Food and Chemical Toxicology*.

[6] Karak T., Bhagat R. M. (2010). Trace elements in tea leaves, made tea and tea infusion: a review. *Food Research International*.

[7] Welz B., Sperling M. (1999). *Atomic Absorption Spectroscopy*.

[8] Waterlow J. C. (1992). *Protein-Energy Malnutrition*.

[9] Classen H. G. (1984). Magnesium and potassium deprivation and supplementation in animals and man: aspects in view of intestinal absorption. *Magnesium*.

[10] Ma J., Folsom A. R., Melnick S. L. (1995). Associations of serum and dietary magnesium with cardiovascular disease, hypertension, diabetes, insulin, and carotid arterial wall thickness: the aric study. *Journal of Clinical Epidemiology*.

[11] WHO (2004). *Vitamin and Mineral Requirements in Human Nutrition: Report of a Joint FAO/WHO Expert Consultation*.

[12] International Agency for Research on Cancer (1990). *IARC Monographs on the Evaluation of Carcinogenic Risks to Humans. Nickel and Nickel Compounds*.

[13] Fleischer M. (1982). New mineral names. *The American Mineralogist*.

[14] World Health Organization (WHO) (1996). Trace elements in human nutrition and health. *A Report of a Re-Evaluation of the Role of Trace Elements in Human Health and Nutrition*.

[15] Vincent J. B. (2000). Elucidating a biological role for chromium at a molecular level. *Accounts of Chemical Research*.

[16] Erikson K. M., Syversen T., Aschner J. L., Aschner M. (2005). Interactions between excessive manganese exposures and dietary iron-deficiency in neurodegeneration. *Environmental Toxicology and Pharmacology*.

[17] Bowman A. B., Kwakye G. F., Hernández E. H., Aschner M. (2011). Role of manganese in neurodegenerative diseases. *Journal of Trace Elements in Medicine and Biology*.

[18] de Maeyer E., Adiels-Tegman M. (1985). The prevalence of anaemia in the world. *World Health Statistics Quarterly*.

[19] International Agency for Research on Cancer (IARC) (1990). Monographs on the evaluation of carcinogenic risks to humans. *Chromium, Nickel and Welding*.

[20] Schlegel-Zawadzka M., Nowak G. (2000). Alterations in serum and brain trace element levels after antidepressant treatment. Part II. Copper. *Biological Trace Element Research*.

[21] World Health Organisation (2004). *Vitamin and Mineral Requirements in Human Nutrition: Report of a Joint FAO/WHO Expert Consultation*.

[22] Shankar A. H., Prasad A. S. (1998). Zinc and immune function: the biological basis of altered resistance to infection. *The American Journal of Clinical Nutrition*.

[23]  Joint FAO/WHO Expert Committee on Food Additives (2005). *Evaluation of Certain Food Additives (Sixty-Third Report of the Joint FAO/WHO Expert Committee on Food Additives)*.

[24] Liva R. (2007). Facing the problem of dietary-supplement heavy-metal contamination: How to take responsible action. *Integrative Medicine*.

[25] Hutton M., Hutchinson T. C., Meema K. M. (1987). Human health concerns of lead, mercury, cadmium and arsenic. *Lead, Mercury, Cadmium and Arsenic in the Environment*.

[26] Szymczycha-Madeja A., Welna M., Pohl P. (2012). Elemental analysis of teas and their infusions by spectrometric methods. *Trends in Analytical Chemistry*.

[27] Shen F.-M., Chen H.-W. (2008). Element composition of tea leaves and tea infusions and its impact on health. *Bulletin of Environmental Contamination and Toxicology*.

